# Is there a difference in the results of the video head impulse test in patients with a nosological diagnosis of Ménière’s Disease and Vestibular Migraine?

**DOI:** 10.1590/2317-1782/e20230359en

**Published:** 2025-01-20

**Authors:** Maria Carolaine Ferreira Aguiar, Gizele Francisco Ferreira do Nascimento, Ana Paula Machado Costa, Lidiane Maria de Brito Macedo Ferreira, José Diniz, Erika Barioni Mantello

**Affiliations:** 1 Programa Associado de Pós-graduação em Fonoaudiologia (Mestrado) – PPgFon, Universidade Federal do Rio Grande do Norte – UFRN - Natal (RN), Brasil.; 2 Departamento de Fonoaudiologia, Universidade Federal do Rio Grande do Norte – UFRN - Natal (RN), Brasil.; 3 Ambulatório de Otorrinolaringologia, Empresa Brasileira de Serviços Hospitalares – EBSERH, Hospital Universitário Onofre Lopes – HUOL, Universidade Federal do Rio Grande do Norte – UFRN - Natal (RN), Brasil.; 4 Ambulatório de Otoneurologia, Departamento de Cirurgia, Universidade Federal do Rio Grande do Norte – UFRN – Natal (RN), Brasil.

**Keywords:** Vertigo, Head Impulse Test, Migraine Disorders, Meniere’s Disease, Dizziness

## Abstract

**Purpose:**

To compare vestibulo-ocular reflex (VOR) gain values, gain symmetry between the semicircular canals (SCCs), and saccadic parameters in patients with a nosological diagnosis of Ménière’s disease (MD) and vestibular migraine (VM).

**Methods:**

Observational, descriptive, cross-sectional, retrospective study, approved by the Research Ethics Committee, under evaluation report number 4.462.519. The study was based on medical record analysis of individuals who underwent the Video Head Impulse Test (vHIT). The sample included medical records of 33 patients, divided into two groups – G1, 18 patients with a nosological diagnosis of VM; G2, 15 patients with MD. The study collected information on age, sex, nosological diagnosis, symptoms, associated comorbidities, and vHIT results. Student's t-test and the linear regression model statistically analyzed the data. The significance level was set at 0.05 (95%).

**Results:**

Females predominated (75.76%), with a mean age of 50.18 years. There was a predominance of normal VOR gain in the VM group (44.44%) and vestibular hypofunction in the MD group (40%). There was no significant difference between the groups’ mean gain per SCC, nor between the groups’ right and left SCCs. G1 had a higher percentage of evident saccades and saccadic dispersion.

**Conclusion:**

Although there was no significant difference in VOR gain in the vHIT between the groups, there was a predominance of vestibular hypofunction in the MD group and normal results in the VM group.

## INTRODUCTION

Dizziness is a common complaint in the global population and its prevalence increases with age^(1)^. Establishing and diagnosing the cause of dizziness is still a challenging task, as it is often based on clinical criteria, guidelines on the topic, and patient symptomatic reports^(2)^. Various etiologies affect body balance and the vestibular system, including Ménière's disease (MD) and vestibular migraine (VM), with a significant portion of diagnoses. Both diseases negatively impact the physical, emotional, and occupational health of their sufferers, causing several impairments in the patient's quality of life^(3)^.

MD is a syndrome characterized by episodes of spontaneous vertigo, accompanied by sensorineural hearing loss, tinnitus, and aural fullness in the affected ear. Its pathophysiology is related to endolymphatic hydrops, an excess of endolymph in the membranous labyrinth that dilates the cochlear duct, saccule, utricle, and semicircular canals (SCCs)^(4)^. In addition to vestibular symptoms, MD can also cause migraine episodes^(3)^.

The latest consensus published by the Bárány Society^(5)^ established some important points for VM diagnosis, such as current or previous history of migraine with or without aura; one or more migraine attacks with at least 50% of vestibular episodes; unilateral, pulsating headache with moderate to intense pain, worsening with physical activities; phonophobia and photophobia; and visual aura not better explained by any other vestibular diagnosis or the International Classification of Headache Disorders. Its pathophysiology involves several neural pathways, including the vestibular nuclei, trigeminal nerve, thalamus, and cortical areas, with simultaneous activation of nociceptive vestibular pathways^(5,6)^.

Several current tests can investigate the causes of dizziness, but none of them fully assess vestibular function^(6)^. Therefore, it is recommended that patients with vestibular symptoms undergo a combination of clinical, functional, and instrumental tests to collaborate toward an accurate diagnosis and define treatment^(6)^.

The Video Head Impulse Test (vHIT) stands out among objective tests for assessing the gain of the vestibulo-ocular reflex (VOR) at high frequency, similar to the physiological stimulation of everyday head movements. Thus, it helps diagnose several vestibular diseases and is recommended, especially when combined with other tests^(7)^.

MD is well documented in the literature. However, there is still no consensus on how it affects vestibular function^(8)^. Likewise, the causes of VM remain uncertain, making diagnosis challenging since it depends on symptomatic characterization^(9)^.

Thus, instrumental vestibular tests can provide important information for the diagnosis, prognosis, and therapeutic monitoring of vestibular dysfunctions such as MD and VM. Considering that both clinical conditions have similar symptoms, this study aimed to compare the values ​​of VOR gain, symmetry between the SCCs, and saccadic parameters in patients with a nosological diagnosis of MD and VM.

## METHOD

This preliminary, primary, observational, descriptive, cross-sectional, retrospective study was approved by the research ethics committee of the Onofre Lopes University Hospital (HUOL) under evaluation report number 4.462.519. The sample was established by reviewing the medical records of patients treated at HUOL’s otorhinolaryngology outpatient clinic from September 2021 to May 2022. The medical records were selected after the patients had duly signed and filled out an informed consent form with an attached data consent form.

The inclusion criteria were adult or older patients, of both sexes, treated at the institution's otoneurology outpatient clinic, with a nosological diagnosis of MD or VM, who underwent the vHIT within 3 months after the first visit to the otorhinolaryngologist when the suspected diagnostic hypothesis was defined. The study excluded medical records of patients with chronic degenerative diseases or tumors in the central nervous system; who had a nosological medical diagnosis of other vestibular diseases (e.g., benign paroxysmal postural vertigo, vestibular neuritis, persistent postural-perceptual dizziness); and registration forms with incomplete data in the electronic medical record.

The study analyzed 102 patient records, but only 33 met the study eligibility criteria. It collected information on age, sex, nosological diagnosis, clinical manifestations, associated comorbidities, and vHIT results.

The vHIT analysis approached VOR gain, gain symmetry between SCCs, and parameters of compensatory, covert, and overt saccades (amplitude, latency, and organization). Normal VOR gain values were those proposed by previous studies^(6,7)^, ranging from 0.8 to 1.20 for lateral canals and from 0.7 to 1.20 for vertical canals; the symmetry between SCCs should be less than 20%. Reduced VOR gain and/or compensatory saccades indicated abnormal examinations^(10)^.

The saccadic dispersion rate was measured with the Perez and Rey Score (PR Score), which measures the rate of compensatory saccade organization as a function of time. It is expressed from 0 (zero) to 100 points – higher scores indicate greater dispersion of compensatory saccades (characteristic of incomplete vestibular compensation), while lower scores indicate greater grouping of saccades (characteristic of the vestibular system closer to complete vestibular compensation), in line with increased VOR gain^(10)^.

Patients were divided into two groups for data analysis, according to the nosological medical diagnosis: Group 1 comprised individuals diagnosed with VM, and Group 2 comprised patients diagnosed with MD.

The data were analyzed using the SAS 9.0 statistical software. The descriptive analysis included absolute and relative frequencies of the qualitative variables and the means of quantitative variables, which underwent normality analysis with the Shapiro-Wilk Test.

Student's t-test for independent data compared two means from unpaired samples (VM and MD Groups) in the inferential analysis. This test requires verifying whether the variances of the two groups are statistically equal and whether the data follow a normal distribution. Generalized linear regression analyzed symmetries between the groups. The linear regression model with mixed effects analyzed the repeated measures for the same individual, considering the right and left sides to obtain the statistical difference in VOR gain comparison, using the classification as a confounding factor (covariate) to categorize the change. This study set the significance level at 0.05 (95%).

It was not possible to perform inferential analysis to compare saccadic parameters due to the low occurrence of saccades per SCC both individually and per study group. Therefore, they underwent descriptive analysis with a comparison of means.

## RESULTS

The sample had 33 individuals, divided into two groups – G1 (54.55%), with 18 patients diagnosed with VM, and G2 (45.45%) with 15 patients with MD. The overall mean age was 50 years, ranging from 18 to 77 years. The mean age per group was 46 years in G1 and 54 years in G2, with no statistical difference between groups (p = 0.1347).

Most individuals (75.76%) were females, while 24.24% were males. In G1, 77.78% of the sample were women, and 22.22% were men; in G2, 73.33% were women, and 26.67% were men.

Vertigo (78.79%), headache (63.64%), and tinnitus (60.61%) were the predominant complaints in the general sample. Systemic arterial hypertension (SAH) was the prevalent comorbidity associated with VM and DM (33.33%), followed by anxiety (12.12%), osteoporosis (12.12%), and diabetes mellitus (6.06%).

Regarding the vHIT parameters analyzed, normal vestibular function prevailed in 44.44% of the G1 sample, while 33.33% of them had vestibular hypofunction, mainly in the anterior SCCs (55.56%), followed by the lateral (33.33%) and posterior (11.11%) canals; in addition, 22.22% had increased VOR gain in either of the lateral canals or bilaterally. In G2, 26.67% of the sample had normal VOR gain, while 40% had SCC hypofunction, mainly affecting the anterior and posterior SCCs (46.15%), with less impact on the lateral canals (7.69%). An increase in VOR gain was also identified in the lateral canals in 33.33% of the cases.

**Figure 1:**
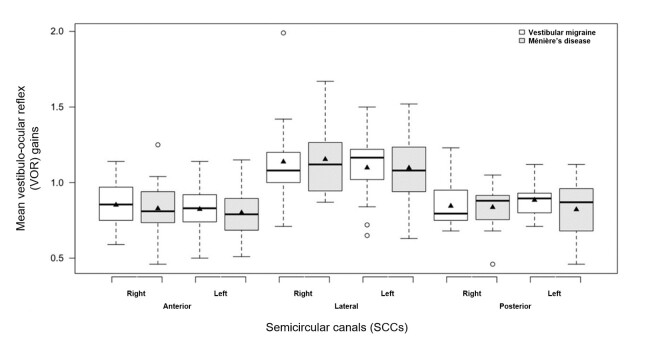
Boxplot comparing mean VOR gains in the anterior, lateral, and posterior right and left SCCs between G1 (vestibular migraine) and G2 (Ménière’s disease)

The mean VOR gain of the general sample (all SCCs) in the vHIT ranged from 0.80 to 1.15. There was no statistical difference in the mean gain of the anterior (p = 0.8419), lateral (p = 0.4566), or posterior (p = 0.6435) SCCs between G1 and G2, regardless of the right and left sides (Figure 1). Figure 1 shows the representative boxplot of the mean gains in each SCC in G1 and G2.

Caption: VOR: Vestibulo-ocular reflex; SCCs: semicircular canals

**Figure 2:**
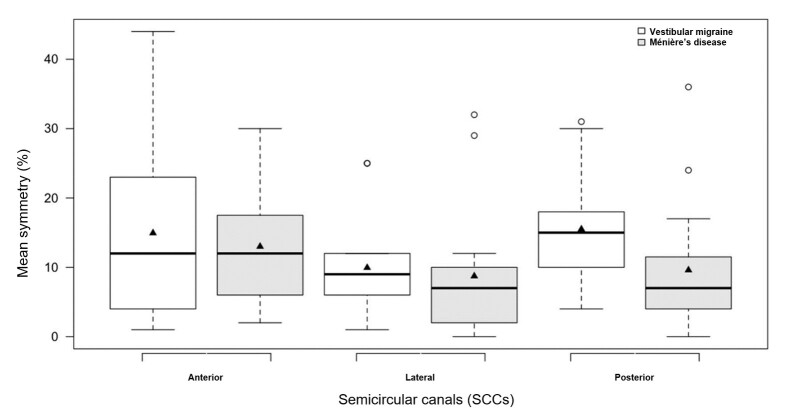
Boxplot comparing symmetry means in the anterior, lateral, and posterior SCCs between G1 (vestibular migraine) and G2 (Ménière’s disease)

The symmetry between the SCCs ranged from 8.73% to 15.50%, with evidence of statistical difference only for the posterior SCCs (p = 0.042) and a greater difference in asymmetry in G1. Figure 2 shows the boxplot representing the mean symmetry of the SCCs between the groups.

Caption: SCCs: semicircular canals

As for saccadic parameters, the mean percentage of compensatory saccades was slightly higher in G1 (28.79%) than in G2 (23.01%), highlighting the greater occurrence of evident saccades in the left lateral SCC. G1 also had higher mean saccadic dispersion rates (PR score) ​​(34.80%) than G2 (24.16%), suggestive of greater dispersion, characteristic of incomplete vestibular compensation.

The SCCs with the highest occurrence of evident saccades in G1 were the left lateral SCC, with 63%; left posterior SCC, with 33%; and left anterior SCC, with 29%. G2 obtained 20% in the left lateral SCC; 16% in the left posterior SCC; and 13% in the left anterior SCC.

## DISCUSSION

Females predominated in both groups in this study, with a mean age of 50 years. The literature also describes such findings, indicating the prevalence of endolymphatic hydrops and VM in this age group^(8,11)^. The greater incidence in females may be related to the worsening of symptoms due to hormonal factors (especially in cases of headache) or the interference of menopause and changes in the menstrual cycle, whether in VM or MD^(11)^.

VM symptoms include visual aura, photophobia, and unilateral throbbing headache. MD symptoms include vertigo, aural fullness, tinnitus, and documented sensorineural hearing loss, especially in low frequencies^(4)^. Many of these signs and symptoms were manifested in both groups in this study, suggesting a probable association between MD and VM^(4,12)^.

Hypertension was the predominant comorbidity in this sample (33.33%) in both groups. This incidence can be justified by vestibular system hypersensitivity due to a circulatory disorder^(13)^.

Regarding the vHIT parameters evaluated, part of the sample had VOR gain within normal limits. This corroborates the sovereignty of clinical diagnosis, especially for VM cases since vestibular tests such as the caloric test (CT), cervical vestibular evoked myogenic potential, and vHIT commonly present normal results^(12)^.

A previous study^(13)^ reported that vHIT in DM diagnosis can detect changes in only 37% of cases, close to the rates of exams affected by vestibular hypofunction in the present study (40%).

The vHIT is less sensitive than CT to detect changes in MD, which can be justified by the fact that the disease mainly damages type II (peripheral) cells and spares type I (central) cells – and vHIT mainly stimulates type I cells, while CT mainly stimulates type II cells^(14)^. Although some studies^(10,13,14)^ found a higher prevalence of changes in MD assessed by CP than by vHIT, this should not be used as the sole parameter to assess changes in vestibular function since this test is limited to investigating gains in lateral SCCs at low frequencies and uses non-physiological stimuli^(13,14)^.

G1 had a higher incidence of hypofunction in the anterior canals, followed by the lateral ones. A literature review^(15)^ showed that vHIT can characterize different VOR gain results in central diseases, varying from gain within the normal criteria, hypofunction restricted to the lateral SCCs, and lower gain values ​​in vertical SCCs than in lateral ones – a result found in the VM group in this study. This diversity of central disease findings is justified by the involvement of the vestibular nerve, vestibular nucleus, or deep cerebellar nuclei that modulate the VOR, confirming the involvement of this reflex and its connections in central diseases^(15)^. Increased sensitivity to sensory stimuli is one of the pathophysiological mechanisms accepted to explain VM^(12)^. Recent studies^(10,15)^ indicate functional vestibular system hypersensitivity in individuals with migraine and describe that these patients would have a lower movement detection threshold, resulting in an exaggerated VOR, suggested by the increased VOR gain in this research.

A study^(16)^ reported more frequent vestibular hypofunction in the posterior SCCs, followed by the lateral ones, in patients with MD. It justified these findings with the chronic course of the disease, resulting in hypofunction progression in the posterior region. The present study likewise found higher vestibular hypofunction rates in the posterior canals in G2 (with MD).

There was a higher percentage of abnormal exams (compatible with vestibular hypofunction) in the MD group (73.33%) than in the VM group (55.55%). This reinforces the literature findings^(3)^, ascribing it to the fact that DM is a peripheral vestibulopathy (which causes changes in the inner ear and, consequently, in the areas investigated by vHIT), while VM has its eventual cause in trigeminovascular activation with an inflammatory response of the intracranial vessels (which affect the inner ear more transiently)^(12,16)^.

The increased VOR gain in the lateral canals, especially in G2 (33.33%), may be related to the period when they were examined – which may have been outside the crisis in part of the sample since MD patients in quiescence are free of signs of vertigo. However vHIT measurements show that VOR gain may be typically increased^(16)^. Vertigo is intense during a crisis, with a decrease in VOR gain^(16)^.

The vHIT can be considered abnormal when it detects a deficit in VOR gain and changes in compensatory saccades^(10)^. G1 in this study had a higher mean percentage of occurrence of evident compensatory saccades and a higher dispersion rate in the lateral canals, characteristic of incomplete vestibular compensation^(14)^.

Despite the technology available for instrumental vestibular system assessment, reaching a DM or VM diagnosis is often challenging. The principles for confirming the suspected diagnostic are commonly based on the findings of clinical otoneurological assessment in combination with the medical history survey.

Since this is a retrospective study, it is important to highlight the small sample size, the duration of the disease, and the difficulty in controlling some variables as limiting factors of this study. The medical record analysis could not identify whether the patients had undergone vHIT outside the period of symptom crisis.

Nevertheless, the study considered the vHIT a valid instrument to confirm the presence of vestibular hypofunction, determine the affected side and SCCs, characterize findings of the central VM etiology (e.g., increased gain), and make inferences about the patients’ vestibular compensation phase. Associating these findings with clinical history contributes to the accurate diagnosis and direction of individualized treatment.

## CONCLUSION

This study considered the vHIT a valid instrument to confirm or rule out vestibular hypofunction. However, it did not find statistical differences in such examination results between VM (G1) and MD (G2), except for posterior SCC asymmetry.
