# Supplementary material for: Is there a difference in the results of the video head impulse test in patients with a nosological diagnosis of Ménière’s Disease and Vestibular Migraine?
Source: Codas. 2025 Jan 20;37(1):e20230359. doi: 10.1590/2317-1782/e20230359en (PMC11758851; doi:10.1590/2317-1782/e20230359en)
Supplement: Translation

# Translation: Existe diferença nos resultados do vídeo teste do impulso cefálico em pacientes com diagnóstico nosológico de Doença de Ménière e Migrânea Vestibular?

**DOI:** 10.1590/2317-1782/e20230359pt

**Authors:** Maria Carolaine Ferreira Aguiar, Gizele Francisco Ferreira do Nascimento, Ana Paula Machado Costa, Lidiane Maria de Brito Macedo Ferreira, José Diniz, Erika Barioni Mantello

**Affiliations:** 1 Programa Associado de Pós-graduação em Fonoaudiologia (Mestrado) – PPgFon, Universidade Federal do Rio Grande do Norte – UFRN - Natal (RN), Brasil.; 2 Departamento de Fonoaudiologia, Universidade Federal do Rio Grande do Norte – UFRN - Natal (RN), Brasil.; 3 Ambulatório de Otorrinolaringologia, Empresa Brasileira de Serviços Hospitalares – EBSERH, Hospital Universitário Onofre Lopes – HUOL, Universidade Federal do Rio Grande do Norte – UFRN - Natal (RN), Brasil.; 4 Ambulatório de Otoneurologia, Departamento de Cirurgia, Universidade Federal do Rio Grande do Norte – UFRN – Natal (RN), Brasil.

**Keywords:** Vertigem, Teste do Impulso da Cabeça, Transtornos de Enxaqueca, Doença de Méniere, Tontura

## Abstract

**Objetivo:**

Comparar os valores de ganho do reflexo vestíbulo-ocular (RVO), simetria de ganho entre os canais semicirculares (CSCs) e parâmetros sacádicos em pacientes com diagnóstico nosológico de doença de Méniere (DM) e migrânea vestibular (MV).

**Método:**

Estudo observacional, descritivo, transversal e retrospectivo, aprovado pelo Comitê de Ética em Pesquisa, sob parecer: 4.462.519. O estudo foi realizado a partir da análise de prontuários de indivíduos que realizaram o vídeo teste do impulso cefálico (vHIT). Foram incluídos prontuários de 33 pacientes, divididos em dois grupos: G1, 18 pacientes com diagnóstico nosológico de MV, G2, 15 pacientes com diagnóstico de DM. Foram coletadas informações sobre idade, gênero, diagnóstico nosológico, sintomas, comorbidades associadas e resultados do vHIT. Os dados obtidos foram analisados estatisticamente, por meio do Teste t-student e Modelo de regressão linear. Foi adotado o nível de significância de 0,05 (95%).

**Resultados:**

O sexo prevalente foi o feminino (75,76%), com idade média de 50,18 anos. Observou- se predomínio do ganho do RVO dentro dos padrões de normalidade para o grupo MV (44,44%) e da hipofunção vestibular para o grupo DM (40%). Não houve diferença significativa entre a média de ganho por CSC, dos grupos, nem entre os CSCs direito e esquerdo, dos grupos. Quanto aos parâmetros sacádicos, o G1 apresentou maior percentual de sacadas evidentes e da dispersão sacádica.

**Conclusão:**

Embora não se tenha encontrado diferença significativa entre o ganho do RVO no vHIT entre os grupos, observou-se predomínio da hipofunção vestibular no grupo DM e de resultados dentro da normalidade no grupo MV.

## INTRODUÇÃO

A tontura é uma queixa comum na população mundial e sua prevalência aumenta com a idade^(1)^. Estabelecer e diagnosticar a causa da tontura ainda é uma tarefa desafiadora, pois é frequentemente baseada em critérios clínicos, *guidelines* da área e relatos sintomáticos do paciente^(2)^. Diversas são as etiologias que afetam o equilíbrio corporal e o sistema vestibular, dentre elas, a doença de Ménière (DM) e a Migrânea Vestibular (MV) representam uma parcela importante dos diagnósticos. Ambas as doenças impactam negativamente a saúde física, emocional e ocupacional do seu portador, acarretam diversos prejuízos na qualidade de vida do paciente^(3)^.

A DM é uma síndrome caracterizada por episódios de vertigem espontânea, acompanhadas da perda auditiva sensorioneural, zumbido e sensação de plenitude aural na orelha afetada. Sua fisiopatologia está relacionada à hidropsia endolinfática, um excesso de endolinfa no labirinto membranoso, o que acarreta a dilatação do ducto coclear, sáculo, utrículo e canais semicirculares (CSCs)^(4)^. Além dos sintomas vestibulares, a DM também pode ocasionar episódios de enxaqueca^(3)^.

No último consenso publicado pela Bárány Society^(5)^ foi estabelecido alguns pontos importantes para o diagnóstico da MV, como: história atual ou anterior de enxaqueca com ou sem aura; uma ou mais crise de enxaqueca com pelo menos 50% de episódios vestibulares; dor de cabeça unilateral, pulsátil e de com sensação de dor moderada a intensa, que se agrava na realização de atividades físicas; fonofobia e fotofobia; aura visual e que não é melhor explicada por outro diagnóstico vestibular ou na classificação internacional das cefaleias. A fisiopatologia envolve várias vias neurais, incluindo os núcleos vestibulares, nervo trigêmeo, tálamo e áreas corticais, com ativação simultânea de vias vestibulares nocioceptivas^(5,6)^.

Atualmente existem vários exames capazes de investigar as causas da tontura. No entanto, nenhum deles avalia integralmente a função vestibular^(6)^. Portanto, recomenda-se que pacientes com sintomas vestibulares sejam submetidos a uma combinação de testes clínicos, funcionais e instrumentais, com o objetivo de colaborar para um diagnóstico preciso e direcionar o tratamento^(6)^.

Dentre os testes objetivos, o vHIT destaca-se como um teste que permite avaliar o ganho do reflexo vestíbulo-ocular (RVO) em alta frequência, semelhante a estimulação fisiológica dos movimentos de cabeça cotidianos, portanto auxilia no diagnóstico de diversas doenças vestibulares e é recomendado, especialmente quando combinado com outros testes^(7)^.

A DM é bem documentada na literatura, no entanto, ainda não existe um consenso sobre como a função vestibular é afetada pela mesma^(8)^. Da mesma forma, as causas da MV permanecem incertas, tornando o diagnóstico desafiador, uma vez que depende da caracterização sintomatológica^(9)^.

Desta forma, os testes vestibulares instrumentais podem fornecer informações importantes para o diagnóstico, prognóstico e monitoramento terapêutico das disfunções vestibulares como a DM e MV e, ao considerar que sintomas semelhantes são observados em ambos os quadros clínicos, o presente estudo tem como objetivo comparar os valores de ganho do RVO, simetria entre os canais semicirculares e parâmetros sacádicos em pacientes com diagnóstico nosológico de doença de Méniere e migrânea vestibular.

## MÉTODO

Estudo preliminar, primário, observacional, descritivo, transversal e retrospectivo, aprovado pelo comitê de ética em pesquisa (CEP) do Hospital Universitário Onofre Lopes (HUOL), sob o número de parecer 4.462.519. A casuística foi estabelecida por meio da revisão dos prontuários de pacientes atendidos no ambulatório de otorrinolaringologia do HUOL, no período de setembro de 2021 a maio de 2022. Para seleção do prontuário, o Termo de Consentimento Livre e Esclarecido (TCLE) estava devidamente assinado e preenchido, bem como o Termo de Compromisso para Uso de Dados (TCUD) anexado.

Os critérios de inclusão adotados foram pacientes adultos ou idosos, de ambos os gêneros, atendidos no ambulatório de otoneurologia da instituição, com diagnóstico nosológico de doença de Ménière ou migrânea vestibular e, que realizaram o exame vHIT em até três meses, após a primeira consulta com o médico otorrinolaringologista, em que se estabeleceu a hipótese diagnóstica. Foram excluídos os prontuários de pacientes com doenças crônicas degenerativas ou tumores no sistema nervoso central; pacientes que apresentavam diagnóstico médico nosológico de outras doenças vestibulares (como por exemplo: vertigem postural paroxística benigna, neurite vestibular, tontura postural-perceptual persistente); ou ainda fichas de registro com dados incompletos no prontuário médico eletrônico.

Foram analisados 102 prontuários de pacientes, porém, apenas 33 destes preencheram os critérios de elegibilidade estabelecidos para o estudo. Coletou-se informações sobre idade, gênero, diagnóstico nosológico, manifestações clínicas, presença de comorbidades associadas e os resultados do vHIT.

Para análise do vHIT, foram considerados os seguintes parâmetros: ganho do RVO, valores de simetria do ganho entre os CSCs e os parâmetros relacionados às sacadas compensatórias, encobertas e evidentes (amplitude, latência e organização). Os valores de normalidade para o ganho do RVO foram os propostos por estudos anteriores^(6,7)^, sendo o ganho do RVO entre 0,8 a 1,20 para os canais laterais e 0,7 a 1,20 para os canais verticais, a simetria entre os CSCs deve ser menor que 20%. A anormalidade no exame pode ser indicada por um ganho do RVO reduzido e/ou presença de sacadas compensatórias^(10)^.

A taxa de dispersão sacádica foi mensurada a partir do *Perez and Rey Score* – Perez e Rey Escore (PR Escore), cujo objetivo é medir a taxa de organização das sacadas compensatórias em função do tempo. É expressa de 0 (zero) a 100 pontos, sendo que pontuações mais elevadas indicam maior dispersão de sacadas compensatórias, característico de compensação vestibular incompleta, enquanto pontuações inferiores indicam maior agrupamento das sacadas, característica do sistema vestibular mais próximo da compensação vestibular completa, em consonância ao ganho aumentado do RVO^(10)^.

Para análise dos dados, os pacientes foram divididos em dois grupos, conforme o diagnóstico médico nosológico, o Grupo 1 composto por indivíduos com diagnóstico de migrânea vestibular e, o Grupo 2, por pacientes com diagnóstico de doença de Méniere.

Os dados foram analisados no *software* estatístico SAS 9.0. A análise descritiva incluiu frequências absolutas e relativas para as variáveis qualitativas, e a média foi usada para as variáveis quantitativa, as quais foram submetidas a análise de normalidade por meio do Teste Shapiro Wilk.

Na análise inferencial utilizou-se o Teste t- student para dados independentes, a fim de comparar duas médias provenientes de amostras não pareadas (Grupos MV e DM). Para a utilização deste teste é necessário testar se as variâncias dos dois grupos são estatisticamente iguais e se os dados seguem distribuição normal. A regressão linear generalizada foi usada para a análise das simetrias, entre os grupos. O modelo de regressão linear com efeitos mistos analisou as medidas repetidas para o mesmo indivíduo, considerando os lados direito e esquerdo para obter a diferença estatística na comparação do ganho do RVO, com a variável de classificação usada como fator de confundimento (covariável), em função de categorizar a alteração. Neste estudo foi adotado o nível de significância de 0,05 (95%).

Não foi possível realizar análise inferencial na comparação dos parâmetros sacádicos, devido à baixa ocorrência das sacadas por CSC, individualmente e por grupo de estudo, por isso, optou-se pela análise descritiva com comparação das médias.

## RESULTADOS

A amostra incluiu 33 indivíduos, divididos em dois grupos, o G1 (54,55%) composto por 18 pacientes diagnosticados com MV e o G2 (45,45%) por 15 pacientes com DM. A média de idade geral foi de 50 anos, variando entre 18 a 77 anos. O G1 teve uma média de idade de 46 anos, enquanto o G2 apresentou média de 54 anos, sem diferença estatística entre os grupos (p=0,1347).

A maioria dos indivíduos (75,76%) eram do gênero feminino, enquanto 24,24% eram do gênero masculino. No G1, 77,78% da amostra era composta por mulheres e 22,22% por homens e no G2 73,33% eram mulheres e 26,67% eram homens.

Quanto à queixa, observou-se predomínio da vertigem (78,79%), cefaleia (63,64%) e zumbido (60,61%) na amostra geral. Quanto às comorbidades associadas à MV e DM, a hipertensão arterial sistêmica (HAS) foi a prevalente (33,33%), seguida de quadros de ansiedade (12,12%), osteoporose (12,12%) e diabetes mellitus (6,06%).

Com relação aos parâmetros analisados no vHIT, no G1, a função vestibular normal prevaleceu em 44,44% da amostra, enquanto 33,33% dos pacientes apresentavam hipofunção vestibular, principalmente para os CSCs anteriores (55,56%), seguido dos canais laterais (33,33%) e posteriores (11,11%), além disso, 22,22% apresentou ganho de RVO aumentado para um dos canais laterais ou bilateralmente. No G2, 26,67% da amostra apresentou ganho do RVO normal, enquanto 40% apresentaram hipofunção dos CSCs, afetando principalmente os CSCs anteriores e posteriores (46,15%), com menor impacto nos canais laterais (7,69%). Foi também identificado aumento do ganho do RVO para os canais laterais, em 33,33% dos casos.

Com relação à média do ganho do RVO, para a amostra geral (todos os CSCs), no vHIT, constatou-se variação de 0,80 a 1,15. Não houve diferença estatística da média de ganho dos CSCs anteriores (p= 0,8419), laterais (p= 0,4566) e posteriores (p= 0,6435), entre G1 e G2, independente dos lados direito e esquerdo (Figura 1). Na Figura 1 visualiza-se o *boxplot* representativo das médias de ganho para cada CSC, entre G1 e G2.

**Figura 1:** *Boxplot* da comparação das médias de ganho do RVO nos CSCs anteriores, laterais e posteriores (direito e esquerdo), entre G1 (Migrânea vestibular) e G2 (Doença de Méniere)

Legenda: RVO: Reflexo vestíbulo ocular; CSCs: Canais Semicirculares

A simetria entre os CSCs variou de 8,73% a 15,50%, com evidência de diferença estatística apenas para os CSCs posteriores (p=0,042) e maior diferença de assimetria no G1. Na Figura 2 visualiza-se o *boxplot* representativo da média de simetria dos CSCs, entre os grupos.

**Figura 2:** *Boxplot* da comparação das médias da simetria nos CSCs anteriores, laterais e posteriores, entre G1 (Migrânea vestibular) e G2 (Doença de Méniere)

Legenda: CSCs: Canais Semicirculares

Quanto aos parâmetros sacádicos, no G1, a média percentual de sacadas compensatórias foi ligeiramente maior (28,79%) que no G2 (23,01%), destacando-se que houve uma maior ocorrência das sacadas evidentes no CSC lateral esquerdo. Quanto a taxa de dispersão sacádica (PR escore), o G1 também apresentou maiores valores de média (34,80%) que o G2 (24,16%), sugestivo de maior dispersão, característico de compensação vestibular incompleta.

Os canais com maior ocorrência de sacadas evidentes foram: canal lateral esquerdo do G1 com 63%; canal posterior esquerdo com 33% e anterior esquerdo com 29%, enquanto o G2 obteve nesses mesmos canais 20% para o lateral esquerdo; 16% para o posterior esquerdo e 13% para anterior esquerdo.

## DISCUSSÃO

No presente estudo observou-se predomínio da amostra, em ambos os grupos, do sexo feminino, com idade média de 50 anos, achados também descritos na literatura que indicam a prevalência da hidropsia endolinfática e da migrânea vestibular nesta faixa etária^(8,11)^. Este maior acometimento no sexo feminino pode relacionar com o agravamento dos sintomas devido fatores hormonais, especialmente nos casos de cefaleia ou, ainda, à interferência da menopausa e modificações do ciclo menstrual, seja na MV ou DM^(11)^.

Os sintomas da MV incluem aura visual, fotofobia com cefaleia latejante unilateral. Os sintomas de DM incluem vertigem com plenitude aural e zumbido com perda auditiva sensorioneural documentada, especialmente nas frequências baixas^(4)^. Nesta pesquisa, muitos desses sinais e sintomas foram manifestados em ambos os grupos, de modo a sugerir uma provável associação da DM e MV^(4,12)^.

Dentre as comorbidades apresentadas, a HAS foi a predominante nesta amostra (33,33%) em ambos os grupos, sua incidência pode ser justificada pela hipersensibilidade do sistema vestibular, diante de um distúrbio circulatório^(13)^.

Acerca dos parâmetros avaliados no vHIT, parte da amostra apresentou ganho do RVO dentro dos limites de normalidade, corroborando o diagnóstico clínico como soberano, especialmente para os casos de MV, visto que, comumente testes vestibulares como a prova calórica, cVEMP e o vHIT apresentam resultados normais^(12)^.

No diagnóstico da DM, um estudo anterior^(13)^ descreveu que o vHIT é capaz de detectar alterações em apenas 37% dos casos, o que se aproximou dos índices de exames alterados por hipofunção vestibular no presente estudo (40%).

A baixa sensibilidade do vHIT em comparação com a prova calórica (PC), para detectar alterações na DM, pode ser justificada pelo fato de a doença lesionar, preferencialmente, as células Tipo II, localizadas na periferia, enquanto as células Tipo I, localizadas na região central, geralmente são poupadas. O vHIT estimula, preferencialmente, as células Tipo I enquanto a PC, as células Tipo II^(14)^. Ainda que, alguns estudos^(10,13,14)^ constataram maior prevalência de alterações na DM, avaliada pela PC quando comparada ao vHIT, essa não deve ser utilizada como único parâmetro para avaliação de alterações da função vestibular, pois trata-se de um teste limitado à investigação do ganho nos CSCs laterais, em baixas frequências e, utiliza estímulo não fisiológico^(13,14)^.

Dentre os achados de hipofunção obtidos no G1, observou-se maior incidência nos canais anteriores, seguido dos laterais. Uma revisão de literatura^(15)^ mostrou que o vHIT pode caracterizar diferentes resultados de ganho do RVO nas doenças centrais que varia desde ganho dentro dos critérios de normalidade, hipofunção restrita aos CSCs laterais ou, ainda valores de ganho nos CSCs verticais inferiores aos laterais, resultado encontrado no grupo com MV, deste estudo. Essa diversidade de achados, nas afecções centrais, é justificada pelo envolvimento do nervo vestibular, núcleo vestibular ou núcleos cerebelares profundos que modulam o RVO, confirmando o acometimento deste reflexo e de suas conexões pelas doenças de origem central^(15)^. O aumento da sensibilidade a estímulos sensoriais é um dos mecanismos fisiopatológicos aceitos para explicar a MV^(12)^. Estudos recentes^(10,15)^ indicam uma hipersensibilidade funcional do sistema vestibular, em indivíduos com migrânea e descrevem que esses pacientes apresentariam um limiar mais baixo para detectar movimentos, resultando em um RVO exagerado, sugerido pelo ganho aumentado do reflexo RVO encontrado nesta pesquisa.

Um estudo^(16)^ descreveu que a hipofunção vestibular dos CSCs posteriores mais frequente, seguido dos laterais, em pacientes com DM e justificaram estes achados devido ao curso crônico da doença, resultando na progressão da hipofunção na região posterior. No presente estudo, também se identificou maiores índices da hipofunção vestibular para os canais posteriores, no G2, com DM.

Houve maior percentual de exames alterados, compatíveis com hipofunção vestibular, no grupo de DM (73,33%) quando comparado ao grupo MV (55,55%), o que reforça os achados na literatura^(3)^, que atribuem o fato da DM ser uma vestibulopatia de origem periférica, que provoca alterações na orelha interna e, consequentemente nas áreas investigadas pelo vHIT, enquanto a MV tem sua eventual causa na ativação trigeminovascular, com resposta inflamatória dos vasos intracranianos, que afetam a orelha interna de forma mais transitória^(12,16)^.

O ganho do RVO aumentado para os canais laterais, especialmente no G2 (33,33%) pode ter relação com o período de realização do exame, que pode ter sido fora da crise, em parte da amostra; visto que, na quiescência os pacientes com DM estão livres de sinais de vertigem, mas as medidas de vHIT mostram que o ganho de RVO pode estar tipicamente aumentado^(16)^. No período de crise, a vertigem é intensa e há uma diminuição no ganho de RVO^(16)^.

O vHIT pode ser considerado alterado quando detectado déficit de ganho do RVO e presença de sacadas compensatórias alteradas^(10)^. Neste estudo, o G1 apresentou maior média percentual de ocorrência de sacadas compensatórias evidentes, bem como maior taxa de dispersão para os canais laterais, parâmetros característicos de compensação vestibular incompleta^(14)^.

Ressalta-se que, apesar da tecnologia disponível para avaliação instrumentada do sistema vestibular, a definição do diagnóstico da doença de Méniere e da migrânea vestibular é muitas vezes desafiadora e, comumente, os princípios para conclusão da hipótese diagnóstica são baseados nos achados da avaliação otoneurológica clínica associados à anamnese.

É importante destacar, como fatores limitantes deste estudo, a amostra reduzida, o tempo de doença e a dificuldade no controle de algumas variáveis, por se tratar de um estudo retrospectivo. Na análise dos prontuários não foi possível identificar se os pacientes realizaram o vHIT fora do período de crise dos sintomas.

Porém, considerou-se o vHIT como um instrumento válido para confirmar a presença da hipofunção vestibular, determinar o lado e quais CSCs foram afetados, caracterizar achados relativos à etiologia central da MV como o ganho aumentado e, ainda, trazer inferências sobre a fase de compensação vestibular dos pacientes avaliados, achados que quando associados à história clínica colaboram no diagnóstico preciso e no direcionamento do tratamento individualizado.

## CONCLUSÃO

Neste estudo, o vHIT foi considerado um instrumento válido para confirmar ou descartar a hipofunção vestibular, porém não observou-se diferença estatística dos achados deste exame entre a migrânea vestibular (G1) e a doença de Méniere (G2), com exceção da assimetria dos canais posteriores.
